# Supplementary material for: Benefit of stereoscopic volume rendering for the identification of pediatric pulmonary vein stenosis from CT angiography
Source: PLOS Digit Health. 2023 Mar 8;2(3):e0000215. doi: 10.1371/journal.pdig.0000215 (PMC9994716; doi:10.1371/journal.pdig.0000215)
Supplement: Attachment — Submitted filename: Response to reviewers.docx [file pdig.0000215.s002.docx]

To the Reviewers:

Thank you very much for your thoughtful and very helpful comments. We believe that we have addressed your comments, and in doing so, significantly improved the quality of this manuscript and results. In addition, we have clarified our methodology and hope that it is now much more understandable.

Reviewer #1: PDIG-D-21-00111 stereoscopic volume rendering CT

Overall: This study reports on assessment of performance using volumetric images either in 2D or 3D mode for detection pediatric pulmonary vein stenosis from CT angiography.

Abstract: Fine as written. The abstract has been updated because of updated data, based on Reviewer 2’s comment 6. (page 3 of tracked changes version)

1)      Line 4: Suggest using untested rather than unproven. This suggestion has been incorporated (page 4, line 31)

Introduction: Overall fine as written in terms of summarizing the problem area.

1)      Could do a better job of reviewing prior literature – there were a number of studies for example by Getty et al. looking at stereoscopic display of breast images. Additional studies have been included for stereoscopy. These escaped an initial literature search as they either fell outside of CT imaging or cardiovascular applications. (page 5, lines 60 to 66)

Methods: A few points require clarification.

1)      Line 83: What were the inclusion & exclusion criteria for including cases in the study? Inclusion and exclusion criteria have been added (page 6, lines 91 to 93).
2)      Line 89: Verify – the gold standard/truth was only determined by a single person? If so this should be noted as a limitation as could be bias. Now page 6, Line98-100: gold standard/ ground truth was determined by a single individual. The use of follow-up operative findings, imaging and clinical charts increased the objectivity of the ground truth. A paragraph has been added to the discussion to acknowledge the limitation (page 18, lines 284 to 287).
3)      Line 96: Data were not data was as data is plural and datum singular. Page 7, line 105: Data has been changed to datum.
4)      Line 98: It is not clear why the images were split into 2 sets rather than having all the observers read all the cases. Page 7: lines 107 to 120: All of the observers read all the cases over 2 sessions. The images were split into 2 sets because it was possible that the first viewing mode would bias the interpretation of the same cases when viewed in the second mode, ie. If all cases were viewed with monoscopic viewing first, it might bias the viewing of the same cases viewed stereoscopically. Although the viewing order could be equalized by distributing equally among the volunteers, the volunteers were sufficiently nonuniform and sporadic that it would have been difficult to divide the volunteers into 2 exactly equal groups. Another reason for 2 sets was that observers only saw a case once during each session. If they read all cases at once, then they would see the same case twice in a session and likely remember their first response. For this reason, we split into 2 sets with a 2 week interval, and added a dummy case, to diminish the memory of the first cases. To make this clearer to readers, we have tried to clarify in the methods, and added 2 figures (Figs. 2A and 2B).
5)      Line 98: If the cases were randomly divided then there was no way to assure equivalence of cases between the 2 groups of cases. Why not randomize with stratification to at least ensure were equal numbers of normal cases in reach group? Page 7, lines 107-120: Although cases were randomly divided, there was equivalence between the 2 groups with stratification having occurred first. This is now described in the methods.
6)      Line 103: Should explain here rather than later the point of these extra 4 cases and why not counted in the analyses. Page 7, lines 118-119: An explanation has been added to explain the attempt to decrease the participants’ recall of cases in the second session.
7)      Line 107: Provide a reference for or explain in more detail what is meant by “direct volume rendering technique”. Page 8, line 124: A reference has been added for direct volume rendering.
8)      Line 113: The readers in the study could not adjust color or opacity? How do you know the single person who set them wasn’t biased in some way? Was this the same person that established truth or another expert? Page 8, line 130: The readers could not adjust color and opacity. The system is based on an open source VTK system, where adjustment of transfer function is not straightforward, and was likely to be a distraction. The lack of color or opacity adjustment is similar to that on commercial systems, where the number of transfer functions is very limited for volume display, so users would not be very familiar with color transfer function adjustment in their normal workflow. In addition, the bias would be the same for monoscopy and stereoscopy as the color transfer function was exactly the same for a given case displayed in both modes. The person who set the transfer function optimized the color and opacity to show the pathology to greatest advantage but this could have been a bias. It was the same person that established ground truth. A phrase has been added to the methods to say that transfer function could not be adjusted.
9)      Seems like a very useful variable to have measured would have been time to render the decisions. Some prior studies showed differences in time using 2D vs stereoscopic 3D – time often ends up being the far more important factor when someone decides whether to use a given display or mode vs another. Do you have timing data you could report? Page 16, lines 227 to 231: Timing data has been reported and full data included in the supporting information. Times were very variable, and seemed to follow no pattern for 2D or 3D, except that they seemed to be more specific to individuals. As mentioned in the discussion, an earlier study that we did with echocardiography, Harake et al, found that time actually increased for 3D as people found more to look at and seemed to look more carefully.
10)     Line 150: How did they point to things? With a cursor? Page 9, line 151: Individuals pointed to things using a cursor. We have added that information in the methods.
Results: A few points require clarification.
1)      Line 164: Need to clarify here & in tables 2 & 3 what types of errors they were for each reader – clearly there are FNs but seems like there are likely FPs as well and this needs to be separated & reported on as could be significant differences in each as a function of 2D vs 3D as well as experience level. Page 9, lines 159 to 161 and Page 10-11, lines 186 to 199. Type 1 and 2 errors have been defined in methods and reported in the results, and also in tables 1 and 2. Type 2 errors are reported in table 3, as type 1 errors were not found to be significant. Please accept our apologies and note that we had to go back to our original data to calculate type 1 errors, and due to a hard drive failure, the loss of data resulted in incomplete data for type 1 errors. In addition, we found 1 more participant, whose data we have included, changing the results slightly (lower p value for type 2 errors).
2)      Line 167: Why using the median here? Were distributions skewed? Page 10-11, lines 186 to 199: We have added the means. Distributions were not particularly skewed but there was the occasional outlier, so we felt that medians were more representative.
3)      Line 168-169: FN or FP errors? Page 10-11, lines 186 to 200: We have reported both type 1 and type 2 errors.
Conclusions: Overall fine in terms of summarizing the results, discussing in a broader context and noting limitations.
1)      Line 194: There are actually more studies on stereoscopic displays in radiology as noted earlier. Page 16, line 238: This observation is acknowledged. The statement has been clarified to specify “cardiovascular imaging”.
2)      Line 243: This is not a limitation! Paired studies are actually preferred as they do use the same images thus eliminating a source of bias/confounding as well as increasing statistical power. Page 19, lines 302 to 309: While the pairing of observations was not a limitation, the fact that observers might remember their first observation in the second session, and be biased was a limitation.
3)      A limitation is however that you used 2 different sets of images for the readers thus actually introducing potential bias/confounds and statistical power so this does need to be noted as a limitation. Page 7, lines 107-120 and figures 2A and 2B: We apologize for the unclear explanation. We did not use 2 different sets of images for the readers. Each reader read all 18 cases, but the order of presentation varied. We have added more explanation in the methods and figures 2A and 2B to make our study design more clear.
4)      The fact that you did not collect timing data should be noted either as a limitation or as a future point to study. Page 16, lines 227 to 231: We did collect timing data and we have reported summarized data. We have chosen not to include a full table, since the data was random. We have included our time data in the supporting information and posted to a data repository. https://doi.org/10.5281/zenodo.6146408
References: A better lit review is required. We have added additional references in the introduction
Tables & Figures: See points above. We have modified and added to our tables and figures.

Reviewer #2: PDIG-D-21-00111

Major comments
1.      The Introduction of the work is somewhat lengthy and might benefit from some degree of synthesis. More so, while a three-dimensional visualization might be especially beneficial in a first-line imaging technique such as ultrasound, computed tomography (CT) already allows image reconstruction to some degree and might perhaps lead to less pronounced advantages in this context. Additionally, CT implies the use of ionizing radiations, whereas increasing the accuracy of ultrasound with CT becoming less necessary might yield further benefits considering the young age of pulmonary vein stenosis patients Page 4 to 6 of version with tracked changes: We have attempted to shorten the introduction while addressing the first reviewer’s comments about increasing the literature review to include other uses of stereoscopy in medical imaging. We have added a statement about ultrasound having limited visibility, therefore CT being required as a second line technique. Unfortunately, 3D visualization of ultrasound would not be helpful in this case because the ultrasound source 3D data is of low quality and very noisy. Our group is heavily invested in 3D ultrasound in many other areas of the body and cardiovascular system as a means of decreasing CT and MRI use, but the pulmonary veins are surrounded by lungs, and hard to fully see well on ultrasound.
2.      Please consider clarifying the nature of the study, as it appears to be mostly on the retrospective side Page 5, line 85:The nature of the study has been clarified to be a retrospective observational study.
3.      Allowing readers to only visualize three-dimensional rendering, with no access to biplanar or multiplanar reconstructions might induce a significant disadvantage on non-stereoscopic visualization of such images, thus introducing a potential source of bias in results from the present work. Indeed, the advantage observed for stereoscopic volume rendering in complex cases might be due to this intrinsic potential source of bias Pages 18 to 19: The limitation of using only 3D rendering without multiplanar reconstructions has been included in the Discussion. It is not a realistic workflow, as physicians would always view the multiplanar reconstructions. Unfortunately, readers could not access the multiplanar reconstructions, as viewing these would have biased the readers and not tested stereoscopic vs monoscopic volume rendering directly. In addition, multiplanar reconstructions can only be viewed in monoscopic display, so we would have been able to perform a pure monoscopic vs stereoscopic comparison.
4.      The authors might consider clarifying exactly how the study sample was collected, and how cases datasets were divided and presented to individual readers, as this might not appear entirely clear from the manuscript. Inclusion and exclusion criteria, along with relevant time intervals ought to be reported thoroughly. A figure might perhaps be beneficial in this context. Page 6, lines 89 to 93: The collection of study cases has been clarified. Inclusion and exclusion criteria and time intervals have been added.

Page 7, lines 107-120 and figures 2A and 2B: Text and two figures have been added to clarify the division of data sets and presentation of cases to participants.

5.      T-tests work under the assumption of data normality, which might not be the case in this work due to the paucity and peculiarity of the study sample. Thus, the authors might consider utilizing non-parametric statistics. Page 9, line 165: The Mann Whitney and Wilcoxon signed rank tests has been used and reported instead of the paired t test.
6.      Please consider adding a clear definition of what constitutes an error, and which types of error might have been encountered by the study readers. More so, please consider reporting the extent of each reader’s individual experience.

Page 9 lines 159 to 161: Definitions of errors have been added to the methods.

Page 10 to 15, lines 186 to 226 and tables 1 and 2: Type 1 and 2 errors and localization errors have been added to the text in the results, and depicted in tables. Please accept our apologies and note that we had to go back to our original data to calculate type 1 errors, and due to a hard drive failure, the loss of data resulted in incomplete data for type 1 errors (only 19 of 24 participants). In addition, we found 1 more participant, whose data we have included, changing the results slightly (lower p value for type 2 errors).Tables 1 and 2 are summaries of the individual’s experiences for the 3 types of errors. Time data has not been reported individually because it was not significant. We have not included case by case individual data in tables as the files are relatively large and cumbersome for a manuscript. However, we have uploaded the additional time data in the supporting information and all of our data is available on a data repository (https://doi.org/10.5281/zenodo.6146408).

Yours sincerely,

Michelle Noga
