# Supplementary material for: Benefit of stereoscopic volume rendering for the identification of pediatric pulmonary vein stenosis from CT angiography
Source: PLOS Digit Health. 2023 Mar 8;2(3):e0000215. doi: 10.1371/journal.pdig.0000215 (PMC9994716; doi:10.1371/journal.pdig.0000215)
Supplement: Attachment — Submitted filename: Response to reviewers Aug 2022.docx [file pdig.0000215.s003.docx]

To the Reviewers:

Thank you very much for your thoughtful and very helpful comments. We believe that we have addressed your comments, and in doing so, significantly improved the quality of this manuscript and results. Responses are in red colored text.

Reviewer #2:

1. The Introduction of the work is still somewhat lengthy and might benefit from some degree of synthesis. More so, whereas ultrasound might not be viable, the authors might consider briefly outlining why other imaging techniques such as magnetic resonance imaging (MRI) would not be viable for such purposes

Response: The introduction has been shortened to 455 words from 566 words. We have added a reference to include MRI as a technique that is used to image the pulmonary veins, although, as stated in the reference, CT has superior spatial resolution and is still considered the gold standard for surgical intervention.

2.    While indeed the addition of multiplanar reconstructions would not allow for a stereoscopic vs monoscopic evaluation, this would have been closer to routine clinical practice, and the authors might have considered testing two different image sets with and without stereoscopic data as a further aid. Indeed, in all clinical settings, readers would have access to monoscopic and multiplanar reconstruction datasets

Response: The routine clinical practice is indeed to have access to multiplanar reconstruction (MPR) and monoscopic volume rendering. However, providing readers with the multiplanar reconstructions would have biased the readers, so that responses might have been based on MPR rather than stereoscopic or monoscopic volume rendering. This limitation was discussed in paragraph 7 of the discussion. The goal of the study was to conduct a pure stereoscopic vs monoscopic evaluation using pulmonary vein stenosis patient imaging as the subject, rather than suggesting a clinical workflow. With the reduction of text in the introduction and an added statement in paragraph 7 of the discussion, we hope that this goal is more clear to the reader.

Reviewer #3:

This paper seeks to explore the advantages of using stereoscopic displays, rather than a monoscopic view of the same scene, when visualizing volume rendered images of the stenotic pediatric pulmonary vein. Through use studies, the authors conclude that in general there is no statistical difference in the ability of users to identify lesions between conventional 3D display and stereo, the users nevertheless reported greater subjective confidence and demonstrated improved accuracy with complex multi-lesion cases of pulmonary vein stenosis. The authors have made considerable improvements since the first version (upon which this reviewer did not comment).

However, I am confused about the motivation of the study. Is there an un-met need that is being addressed?

Response: The motivation for the study is to investigate whether stereoscopic display results in any improvement in diagnosis and spatial orientation compared to regular monoscopic display. Volume rendering is commonly used in clinical practice for more complex clinical cases, and stereoscopic display is requested in our institution for the most complex cases. However, there is little objective data to support this labor intensive practice.

From my viewpoint the study would carry more weight if it compared multi-planar analysis of the images with both imaging modalities, and in addition to diagnostic accuracy, recorded both the time and cognitive load experienced by the observers.

Response: We elected not to use multiplanar analysis in our study, because it was possible that volunteers could entirely rely on the multiplanar reconstructions, and disregard volume rendering completely. Since the goal of the study was to compare stereoscopy to monoscopy, not to elucidate the best way to make a diagnosis of pulmonary vein stenosis in clinical practice, the only way to remove the effect of viewing multiplanar reconstructions was to remove them entirely. We acknowledge in the discussion that this is not the normal clinical practice. Time data was recorded and is in the supplemental data and summarized in the results. Time data was extremely random, and was not recorded for the last 3 participants. Cognitive load was not recorded as it was difficult to quantify.

While I understand that Lee et al in 2013 demonstrated the superiority of 3D visualization (not stereo) to multi-planar, it would be helpful to re-enforce their findings with your own. It would also have been interesting to have each user comment on whether the analysis of 3D images was more intuitive and ergonomic that scrolling through the 3D dataset via 2D planes. One feasible scenario would be to employ the 3D visualization mode to rapidly identify suspected locations of stenoses and then to verify them on the basis of multi-planar visualization.

Response: The goal of the study was to study stereo vs monoscopic visualization, and a comparison to multiplanar reconstruction would have added another arm to the study, necessitating at least 3 viewing sessions. Because the users were busy physicians and surgeons, a commitment for 3 viewing sessions and at least 3 hours of time per individual would not have been tenable. Therefore, the ergonomic comparison could not be performed. Certainly, this would be a possible suggestion in real life clinical practice to advocate rapid 3D mode for identification and then confirmation with multiplanar reconstruction.

I note that the quality of the image displayed in the paper seems to be far from state of the art by today’s standards.  The images displayed in the cited paper Lee et al. dealing with a similar topic appear to be of much higher quality. State of the art, at least in the Siemens world, involves “cinematic rendering”.

Response: The image quality is certainly below the current state of the art. The images were obtained in 2013 to 2015, and the study carried out from 2015 to 2018. CT technology has improved over the last several years. However, because the study used the same volume rendering for the 2D and 3D display, the image quality was controlled for. Volume rendering was carried out using a homegrown system based on VTK, as no commercial software for stereoscopic volume rendering was available at the time of the study.

It would be helpful if the locations of the stenoses in  Figs 1 and 2 were identified, and also to comment on the nature of the displayed images – they appear to be very different.  Several examples of lesions depicted for different patients would be more helpful than the image of two individuals looking at a screen.

Response: The stenosis in Figure 1 has been identified by an arrow. Figure 2 is a diagram to clarify the study design. Figure 3 is an image of the system for illustrative purposes only, to show the stereoscopic system, but has been removed to avoid confusion. Additional examples of lesions for different patients were not included as the authors believed that additional images would detract from the focus of the manuscript.

While I agree that stereoscopy  seems as if it would be a more natural modality for viewing the 3D images, it is mostly of value when assessing relative depth of objects. I may be missing something here, but I do not believe that depth assessment is actually being employed in the identification of stenoses.

Response: While identification of stenoses is one of the tasks measured in this study, identification of location was another crucial task. If stenosis was recognized, but the vessel was incorrectly identified, this result was recorded as an error. Similarly, readers were asked to identify specific vessels to judge their degree of spatial orientation. Depth perception likely aids in the problem of disorientation while looking at volume rendered images.

Another aspect that I would like to see discussed more fully is the nature of the manipulations the users accessed during their studies. It is mentioned that the users were able to rotate and scale the images, and if so, does not the rotation also act to provide a similar experience to stereo with respect to identification of depth cues. In fact , in 1991,  Sollenberger and Milgram in “A Comparative Study of Rotational and Stereoscopic Computer Graphic Depth Cues”  <https://doi.org/10.1177/154193129103502007>  concluded that rotational displays did a better job than stereo alone (but that the performance was enhanced when using a stereo display with rotation). It would seem therefore that the act of manipulation cannot be teased out from the stereoscopic visualization, and may be the reason that there is so little difference in the results. It would perhaps have been more telling if 4 conditions had been analyzed: Static 3D, Rotating 3D, Static Stereo, Rotating Stereo.

Response: We have added a paragraph in the discussion about the user manipulation and referenced the Sollenberger article. Sollenberger and a few other others did conclude that manipulation does enhance depth cues and perception for simple nonmedical objects, with a greater effect than stereoscopy alone. By allowing manipulation for both monoscopy and stereoscopy, we did control for the act of manipulation. While it would have been interesting to analyze static, rotating, stereo and monoscopic, it would have significantly increased the number of sessions required to complete the study. Such an increase would likely have made the study untenable, considering the nature of the volunteers for this study.

The statement that users performed better overall  with stereoscopy in the complex cases (>= 3 lesions) (p=.03) is curious, since from my reading of the tables, in neither group alone (p=0.12; 0 .11, for trainees and experts respectively) was this the case.  Also given that there were only 6 cases in this category I don’t think any conclusions can regarding differences between mono and stereo  can be made.
Response: The 6 complex cases accounted for one third of the test cases, however they accounted for 64% of monoscopic errors and 58% of stereoscopic errors. Our interpretation of the data was that we should have had a higher proportion of complex cases in order to see an overall difference. The most likely reason that neither the trainee or expert groups had a significant difference for complex cases was that both of these groups were small (13 and 11) so we would have needed a massive difference to see any statistical significance. However, the 2 groups performed similarly, so when combined to a more reasonable group of 24 volunteers, the difference in performance on complex cases reached statistical significance.

Incidentally, in the tables, there is no point in expressing p-values to more than 2 dec places.

Response: p-values have been reported to 2 decimal places.

I believe Line 190 should read “There were 6 type 1 errors…”

Response: We apologize. This sentence has been corrected.

In the supplementary data, the file Type2_and_localization _errors.csv seems to be a duplication of the Time.csv file.

Response: We apologize. This was an error, and has been corrected.

Yours sincerely,

Michelle Noga
